# Supplementary material for: The reversal in the cryptocurrency market before and during the Covid-19 pandemic: Does investor attention matter?
Source: PLoS One. 2024 Nov 27;19(11):e0304377. doi: 10.1371/journal.pone.0304377 (PMC11602072; doi:10.1371/journal.pone.0304377)
Supplement: S1 Appendix — (DOCX) [file pone.0304377.s001.docx]

**Appendix A: Market capitalization and market share of the top 20 largest cryptocurrencies on 1st Apr 2021.**

**Unit: Billion USD**

| Name | ID Code | Market capitalization | Market share |
| --- | --- | --- | --- |
| Bitcoin | BTC | 1,100 | 57.59% |
| Ethereum | ETH | 225 | 11.78% |
| Binance coin | BNB | 49 | 2.57% |
| Tether | USDT | 41 | 2.15% |
| Cardano | ADA | 38 | 1.99% |
| Polkadot | DOT | 34 | 1.78% |
| XRP | XRP | 26 | 1.36% |
| Uniswap | UNI | 15 | 0.79% |
| Litecoin | LTC | 13 | 0.68% |
| THETA | THETA | 13 | 0.68% |
| Chainlink | LINK | 12 | 0.63% |
| USD Coin | USDC | 11 | 0.58% |
| Bitcoin cash | BCH | 10 | 0.52% |
| Filecoin | FIL | 10 | 0.52% |
| Stellar | XLM | 9 | 0.47% |
| Dogecoin | DOGE | 7 | 0.37% |
| TRON | TRX | 7 | 0.37% |
| Solana | SOL | 5 | 0.26% |
| VeChain | VET | 5 | 0.26% |
| EOS | EOS | 5 | 0.26% |
| Total market capitalization: | | 1,910 |  |

Note: Data is collected from https://coinmarketcap.com/charts/#market-cap.
