# Supplementary material for: The reversal in the cryptocurrency market before and during the Covid-19 pandemic: Does investor attention matter?
Source: PLoS One. 2024 Nov 27;19(11):e0304377. doi: 10.1371/journal.pone.0304377 (PMC11602072; doi:10.1371/journal.pone.0304377)
Supplement: S2 Appendix — (DOCX) [file pone.0304377.s002.docx]

**Appendix B. Variable Definition**

| **Variable** | **Definition** |
| --- | --- |
| RET | The daily return of cryptocurrency i on day t is defined as R_i,t_ = ((Price_i_,_t_-Price_i_,_t-1_)/Price_i_,_t-1_) *100 |
| REV | Following Kozlowski *et al.* (2021), the reversal in day t is the cryptocurrency i returns over day t-1. |
| TURN | Kozlowski *et al*. (2021) calculate the total number of Cryptocurrencies outstanding divided by trading volume over the previous day. The formula is:  Turnover = (Trading volume/Cryptocurrencies outstanding) *100 |
| SZ | Kozlowski *et al.* (2021), the size for each cryptocurrency is the logarithm of a crypto's market value at the end of day t. |
| Delta SVI | Following Subramaniam and Chakraborty (2020) to calculate the daily log change of SVI of Cryptocurrency i on day t as ∆SVI_i_,_t_ = ln (SVI_i_,_t_)-ln (SVI_i_,_t-1_) |
| TVOL | Following Bali *et al.* (2011), the total volatility of cryptocurrency i in month d is defined as the standard deviation of daily returns within month d:  $\mathrm{TVOL}_{i, d}=\sqrt{Var({Ret}_{i,t})}$ |
| TSKEW | Following Bali *et al*. (2011), The total Skewness of Cryptocurrency i for month d is computed using daily return within the year y:  ${TSKEW}_{i, d}=\frac{1}{D_{y}}\sum_{t=1}^{D_{y}} \left( \frac{R_{i,t}-\mu_{i}}{\partial_{i}} \right)^{3}$ |
|  | Where D_y_ is the number of trading days in year y, R_i,t_ is the return on cryptocurrency i on day t, µ_i_ is the mean of cryptocurrency returns i in year y, and ∂_i_ is the standard deviation of cryptocurrency returns i in year y. |
