# Supplementary material for: The reversal in the cryptocurrency market before and during the Covid-19 pandemic: Does investor attention matter?
Source: PLoS One. 2024 Nov 27;19(11):e0304377. doi: 10.1371/journal.pone.0304377 (PMC11602072; doi:10.1371/journal.pone.0304377)
Supplement: Attachment — Submitted filename: Review report PONE.docx [file pone.0304377.s004.docx]

Overall the author has done exciting research by investigating the Cryptocurrency Market Before and During the COVID-19 Pandemic. The authors have conducted an in-depth analysis of the prior research and attempted to address the issues.

Nevertheless, the paper can address the following major issues, before publication.

- In the introduction section the author(s) has to add the motivation part and the findings of the study, which will give a clear understanding of the study. Please have a look and change accordingly.
- The review of the literature section should be systematically arranged. The author(s) has missed some relevant literature on COVID-19 and crypto as suggested. They might add them to the literature.
- Proper justification is required for taking the data period from January 2016 to April 2021.
- The market cap of crypto is changing day by day. How the author measures the market cap, while considering time series of study from 2016 to 2021, justify. and the total market cap of all crypto is shown around 2.09 trillion. Is it point estimation or random?
- As the data set is very long, it is better to check the structure breaks of the data.
- How the author(S) has chosen the small and medium-cap crypto? Is there any criterion?
- How the author(s) has chosen the lead and lag variables?
- The author(s) has used methodological contribution as FE and 2SLS model. What is the justification?
- The sub-sample regression model based on which methodology. Make it clear.
- The author may use a robustness study.
- The author(S) has to add other relevant policy suggestions part into this study.

Sahoo, P. K. (2021). COVID-19 pandemic and cryptocurrency markets: an empirical analysis from a linear and nonlinear causal relationship. *Studies in Economics and Finance*, *38*(2), 454-468.

Sahoo, P. K., Sethi, D., & Acharya, D. (2019). Is bitcoin a near stock? Linear and non-linear causal evidence from a price–volume relationship. *International Journal of Managerial Finance*, *15*(4), 533-545.

Sahoo, P. K., & Rath, B. N. (2022). COVID-19 pandemic and bitcoin returns: Evidence from time and frequency domain causality analysis. *Asian Economics Letters*, *4*(Early View).
